# COVID-19 Associated Neurological Manifestation

**DOI:** 10.7759/cureus.33712

**Published:** 2023-01-12

**Authors:** Adnan A Mubaraki, Ohoud Alghamdi, Shatha K Al-halabi, Amal Almoutiri, Kholoud Alhasani, Raghad Alsherbi

**Affiliations:** 1 Department of Medicine, College of Medicine, Taif University, Taif, SAU

**Keywords:** coronavirus, persistent symptoms in covid-19, covid-19 presentation, atypical presentation of covid-19, neurological manifestation, covid-19

## Abstract

Background: At the end of 2019, COVID-19 was first detected in Wuhan. In March 2020, COVID-19 became a pandemic globally. Saudi Arabia registered the first case of COVID-19 on March 2, 2020. This research aimed to identify the prevalence of different neurological manifestations of COVID-19 and to assess the relation of the severity, vaccination state, and continuity of symptoms to the occurrence of these symptoms.

Methods: Cross-sectional retrospective study was done in Saudi Arabia. The study was conducted on previously diagnosed COVID-19 patients by random selection using a predesigned online questionnaire to collect data. Data was entered through Excel and analyzed through SPSS version 23.

Results: The study showed that the most common neurological manifestations in COVID-19 patients are headache (75.8%), changes in sense of smell and taste (74.1%), muscle pain (66.2%), and mood disturbance (depression, anxiety) (49.7%). Whereas other neurological manifestations such as weakness of the limbs, loss of consciousness, seizure, confusion, and vision changes are significantly associated with older individuals, this may lead to increased mortality and morbidity in these patients.

Conclusion: COVID-19 is associated with many neurological manifestations in the population of Saudi Arabia. The prevalence of neurological manifestations is similar to many previous studies, where acute neurological manifestations such as loss of consciousness and convulsions are seen more in older individuals which may lead to increased mortality and worse outcomes. Other self-limited symptoms such as headache and change in smell function i.e., anosmia or hyposmia were more pronounced in those <40 years. This mandates more attention to elderly patients with COVID-19, to early detect common neurological manifestations associated with it, and to apply preventive measures known to improve the outcome of these symptoms.

## Introduction

By end of 2019, COVID-19 was first detected in Wuhan, China [1]. Pneumonia cases started to rise in China due to an undetermined etiology.

On March 2, 2020, the first case of COVID-19 was registered in Saudi Arabia. Since then, the number of cases has been increasing gradually on daily basis, from January 3, 2020, to January 6, 2023; there have been 827,128 confirmed cases of COVID-19 with 9,526 deaths [2,3].

COVID-19 mainly targets the cardiorespiratory system causing cough and fever [4]. The prevalence of neurological manifestations of other coronavirus infections i.e., severe acute respiratory syndrome (SARS) and the Middle East Respiratory Syndrome coronavirus (MERS-CoV) is not well determined [5,6]. The possible association of neurological manifestation with COVID-19 has been described in different systematic reviews [7]. Neurological symptoms of COVID-19 can range from self-limiting (headache, olfactory and/or gustatory dysfunction, dizziness) to more severe ones (disturbed level of consciousness, cerebrovascular accidents, seizures, and rhabdomyolysis) [8,9].

The incidence and characteristics of neurological complications are unclear. Different neurological symptoms had been frequently reported in a cohort of COVID-19 patients. These include meningoencephalitis, cerebrovascular accidents, encephalopathy, seizures, acute peripheral and cranial neuropathies dysgeusia, and olfactory dysfunction [10].

Pinna et al. reported a high prevalence of mood, and post-traumatic stress disorders in COVID-19 patients. Other symptoms include weakness and cognitive dysfunction [11].

Due to the lack of clear and specific symptoms, clinical diagnosis of these patients can be challenging, more so in the absence of fever and respiratory symptoms. Patients with COVID-19 may ignore or be unaware of their illness. Fast recognition and early treatment of these neurological complications are crucial [11].

Due to the minimal data on the prevalence of neurological manifestations of COVID-19, this research was intended to identify the prevalence of different neurological manifestations associated with COVID-19 and the relation of those manifestations to the severity of the disease and the status of vaccination and to better understand different COVID-19-related neurological symptoms and provide an early diagnosis and treatment.

## Materials and methods

This is a cross-sectional retrospective study. The data was collected from all regions of Saudi Arabia. It was conducted between October 2021 and January 2022. The study was conducted on the previously diagnosed COVID-19 patients by random selection using an internet-distributed survey through a data collector with a sample size of 2070 and confidence interval of 95% and a marginal error of 2.15 in the total of infected patients with COVID-19 at the time of study start of 539740. Calculated using an internet-based population calculating software.

Inclusion criteria were to include anyone older than 15 years of age, living in Saudi Arabia, medically free, and with a history of COVID-19 infection confirmed by reverse transcription-polymerase chain reaction (RT-PCR). Those who did not want to participate and/or were less than 15 years of age, and/or with a chronic neurological disease were excluded.

A predesigned online questionnaire was used to collect data. This survey was concluded from other research and included questions about different neurological symptoms such as headache, change in sense of smell and taste, muscle pain, weakness in the limbs, change in peripheral sensation, loss of consciousness, seizure, confusion, any vision change, imbalance or gait disturbance, cerebrovascular accident (stroke), difficulty in swallowing, mood disturbance. We added some questions including demographic data (age, gender, and region), previous neurological disorders, and chronic diseases such as anemia, hypothyroidism, migraine, hypertension, diabetes, and stroke, as these diseases may cause similar neurological symptoms to the COVID-19 associated neurological symptoms. Continuity of symptoms after resolution of COVID-19, COVID-19 vaccine, and history of hospitalization to assess the relation of the severity and vaccination state to the presence of these symptoms. Data was entered through Excel version 16.0.6742.2048 and all statistical analysis was done using Statistical Product and Service Solutions (SPSS) (IBM SPSS Statistics for Windows, Version 23.0, Armonk, NY). P value <0.05 is considered significant.

## Results

**Figure 1:**
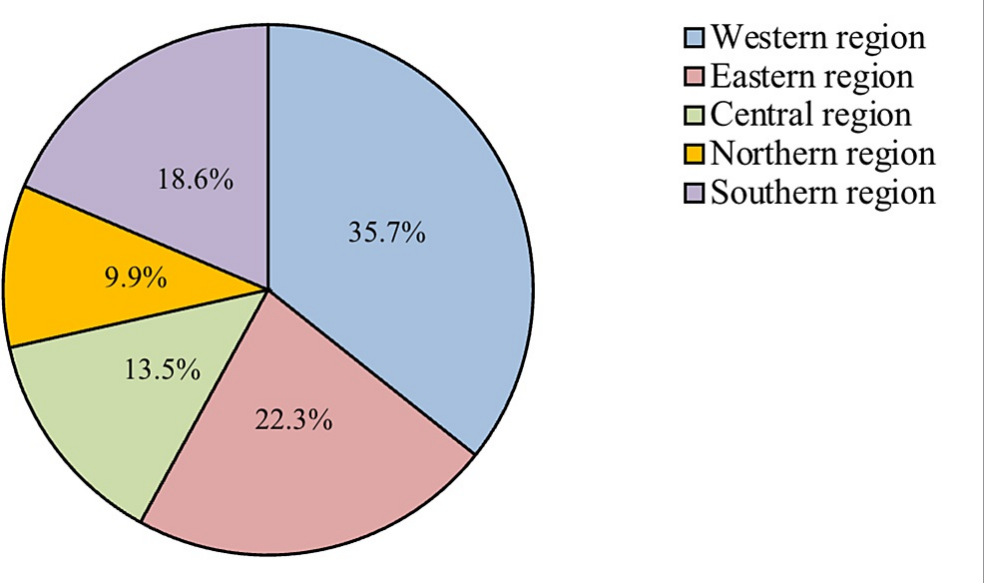
Regions of the study participants (n=2,287)

The 2287 participants who have been diagnosed with COVID-19 were divided demographically into 55.4% females and 44.6% males. The age group of most participants (79.1%) was (15-39 years old) (Table 1). The participants belonged to different regions in the Kingdom of Saudi Arabia, but the percentage of the western region (35.7%) and the eastern region (22.3%) was the highest (Figure 1).

**Table 1:** Demographic characteristics of the participants (n=2,287)

Variable	Frequency	Percent
Gender		
Male	1020	44.6%
Female	1267	55.4%
Age		
15-39	1809	79.1%
40-59	413	18.1%
60 and above	65	2.8%

Patients who reported headache and change in sense of smell and taste as two major neurological manifestations of COVID-19 were 1734 patients (75.8%) and 1695 patients (74.1%) respectively. And there was also a large percentage of the participants who reported having muscle pain and mood disturbance (depression, anxiety) as neurological manifestations of COVID-19, 1515 patients (66.2%) and 1137 patients (49.7%) respectively (Table 2). A total of 19.5% of the vaccinated patients were hospitalized while 80.5% were not. And, 14.1% of the study participants who did not receive the COVID-19 vaccine were hospitalized and 85.9% were not hospitalized (Table 3).

**Table 2:** Prevalence of different neurological manifestations of COVID-19

Neurological symptoms associated with infection with the COVID-19 virus	Yes	No
Headache	1734 (75.8%)	553 (24.2%)
Change in sense of smell and taste	1695 (74.1%)	592 (25.9%)
Muscle pain	1515 (66.2%)	772 (33.8%)
Weakness in the limbs	792 (34.6%)	1495 (65.4%)
Change in peripheral sensation (hand and foot)	513 (22.4%)	1774 (77.6%)
Loss of consciousness	400 (17.5%)	1887 (82.5%)
Seizure	315 (13.8%)	1972 (86.2%)
Confusion	464 (20.3%)	1823 (79.7%)
A vision change	531 (23.2%)	1756 (76.8%)
An imbalance or gait disturbance	682 (29.8%)	1605 (70.2%)
Cerebrovascular accident (stroke)	265 (11.6%)	2022 (88.4%)
Difficulty in swallowing	787 (34.4%)	1500 (65.6%)
Mood disturbance (depression, anxiety)	1137 (49.7%)	1150 (50.3%)

**Table 3:** COVID-19 vaccine and history of hospitalization

COVID-19 vaccine	History of hospitalization	
	Hospitalized	Not hospitalized
Vaccinated (n=921)	180 (19.5%)	741 (80.5%)
Not vaccinated (n=1366)	193 (14.1%)	1173 (85.9%)

**Figure 2:**
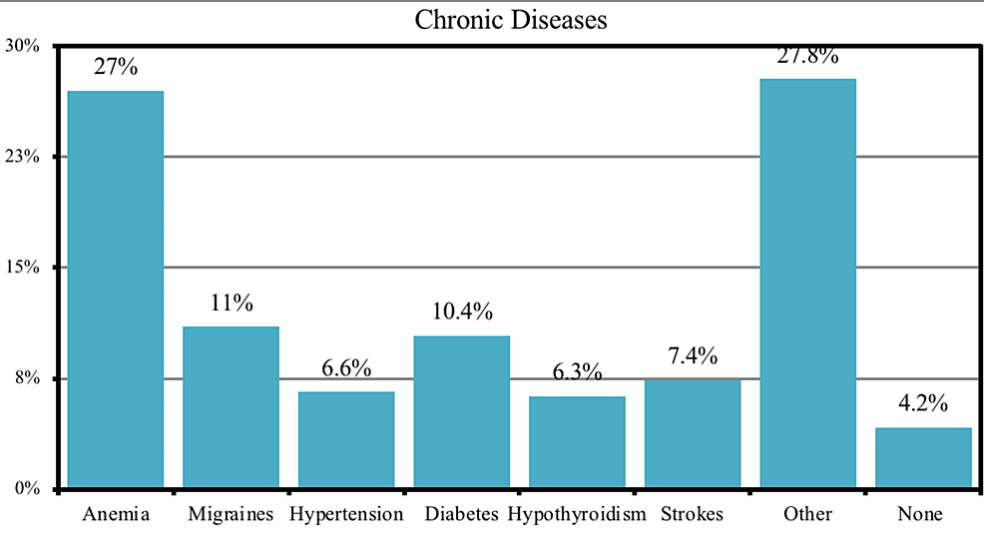
Frequency of chronic diseases among the participants (n=2,287)

**Figure 3:**
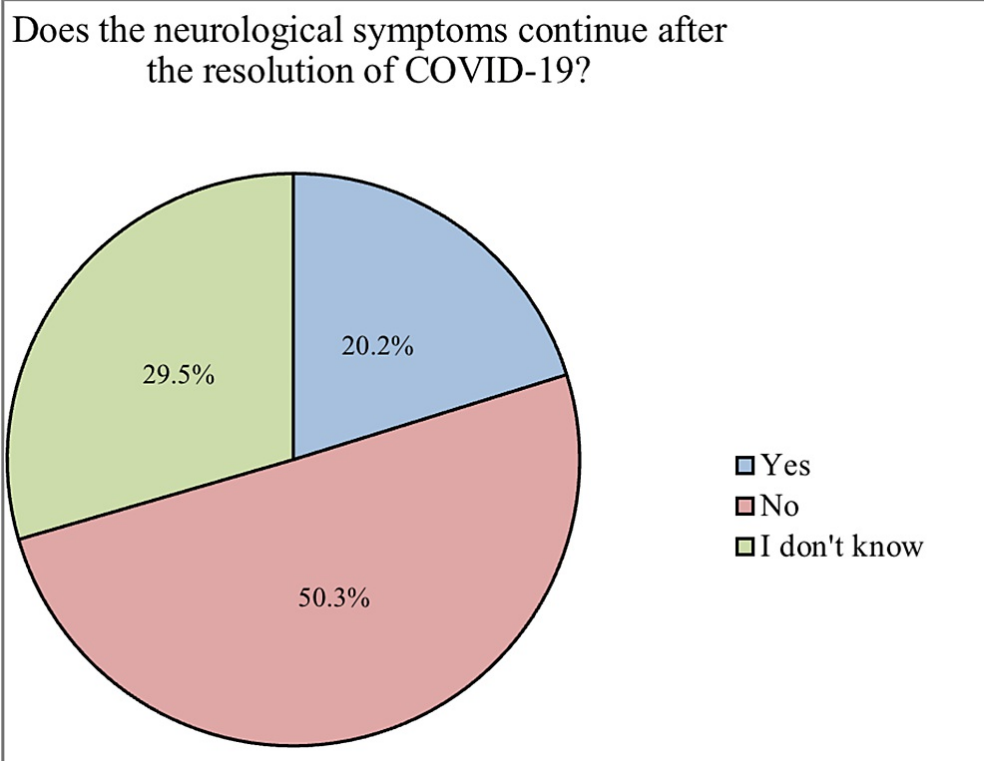
Continuity of neurological symptoms after resolution of COVID-19 (n=2,287)

Most of the participants suffered from anemia (27%) followed by migraines (11%), diabetes (10.4%), and strokes (7.4%) while 27.8% suffered from other chronic diseases such as glucose-6-phosphate dehydrogenase (G6PD) deficiency, allergy, obesity liver diseases, and non-specific rheumatologic condition (Figure 2). 50.3% of patients reported that neurological symptoms did not persist with them after the resolution of COVID-19 (Figure 3). The results showed a significant association (p-value <0.05) between vaccinated patients (40.2%) and the prevalence of most of the neurological symptoms associated with COVID-19, except for the change in the sense of smell and taste, where the prevalence of neurological symptoms associated with COVID-19 in those who were vaccinated was higher than who have not been vaccinated. In addition, there is also a significant association between patients infected with COVID-19 who were admitted to the hospital and the prevalence of neurological symptoms associated with COVID-19, except for the following symptoms (change in the sense of smell and taste, and muscle pain) (Table 4).

**Table 4:** Association between vaccination, hospitalization, and presence of COVID-19-associated neurological symptoms

Neurological symptoms	Vaccination			Hospitalization		
	Yes (%)	No (%)	P value	Yes (%)	No (%)	P value
1. Headache	80.8	72.5	< 0.001	81	74.8	0.011
2. Change in sense of smell and taste	73.7	74.4	0.726	71	74.7	0.139
3. Muscle pain	69.8	63.8	0.003	68.9	65.7	0.236
4. Weakness in the limbs	41.7	29.9	< 0.001	55	30.7	< 0.001
5. Change in peripheral sensation	28	18.7	< 0.001	52.3	16.6	< 0.001
6. Loss of consciousness	21	15.2	< 0.001	50.9	11	< 0.001
7. Seizure	18.1	10.8	< 0.001	41.8	8.3	< 0.001
8. Confusion	25.5	16.8	< 0.001	47.2	15	< 0.001
9. A vision change	29.3	19.1	< 0.001	53.6	17.3	< 0.001
10. An imbalance or gait disturbance	37	25	< 0.001	52.5	25.4	< 0.001
11. Cerebrovascular accident (stroke)	16.9	8	< 0.001	43.2	5.4	< 0.001
12. Difficulty in swallowing	42.5	29	< 0.001	58.7	29.7	< 0.001
13. Mood disturbance	56.8	44.9	< 0.001	64.3	46.9	< 0.001

All neurological symptoms associated with COVID-19 were significantly correlated with their continuation after recovery from COVID-19 disease, as the prevalence of neurological symptoms associated with COVID-19 in those whose symptoms were persistent after recovery from COVID-19 was higher than those with symptoms resolved after the recovery from COVID-19. In addition, there is also a significant association between COVID-19 patients with chronic diseases and the prevalence of COVID-19-related neurological symptoms, except for the following symptoms (change in sense of smell and taste and muscle pain) (Table 5).

**Table 5:** Association between continuity of symptoms after the resolution, presence of chronic diseases, and presence of COVID-19-associated neurological symptoms

Neurological symptoms	Continuity of symptoms after resolution				Presence of chronic diseases		
	Yes (%)	No (%)	I don’t know	P value	Yes (%)	No (%)	P value
1. Headache	76.2	73	80.4	0.001	77.2	72.3	0.015
2. Change in sense of smell and taste	81.2	75.4	67.1	< 0.001	73.4	75.9	0.215
3. Muscle pain	73.2	61.7	69.3	< 0.001	67.2	63.8	0.131
4. Weakness in the limbs	47.8	28.8	35.6	< 0.001	39.4	22.3	< 0.001
5. Change in peripheral sensation	34.2	17.2	23.3	< 0.001	27.7	8.8	< 0.001
6. Loss of consciousness	29	12	19	< 0.001	21.9	6	< 0.001
7. Seizure	25.1	8.6	14.8	< 0.001	17.6	3.8	< 0.001
8. Confusion	34.8	14.4	20.3	< 0.001	23.9	10.8	< 0.001
9. A vision change	40	15.4	25	< 0.001	27.7	11.6	< 0.001
10. An imbalance or gait disturbance	44.2	22.4	32.6	< 0.001	34	19	< 0.001
11. Cerebrovascular accident (stroke)	23.4	6.3	12.6	< 0.001	15.5	1.4	< 0.001
12. Difficulty in swallowing	44.2	29.7	35.9	< 0.001	39.4	21.5	< 0.001
13. Mood disturbance	61.7	43.5	52.1	< 0.001	53.4	40.3	< 0.001

Age group and the prevalence of headache, change in sense of smell and taste, muscle pain, weakness of the limbs, loss of consciousness, seizure, confusion, and a visual change showed also significant correlation as COVID-19-associated neurological symptoms are more associated with the age group( ≥ 60 years) except for muscle pain which was associated with the age group (40-59 years) and headache and change in sense of smell and taste which are associated with age group (15-39 years). The correlation was significant also between gender and the prevalence of muscle pain, loss of consciousness, seizure, an imbalance or gait disturbance, cerebrovascular accident (stroke), and mood disturbance. Prevalence of muscle pain, an imbalance or gait disturbance, and mood disturbance were higher among females while the prevalence of loss of consciousness, seizure, and cerebrovascular accident (stroke) was higher among males (Table 6).

**Table 6:** Association between age, gender, and presence of COVID-19-associated neurological symptoms

Neurological symptoms	Age group (Years)				Gender		
	15-39 (%)	40-59 (%)	≥ 60 (%)	P value	Male (%)	Female (%)	P value
1. Headache	77.1	71.7	66.2	0.012	75.4	76.2	0.668
2. Change in sense of smell and taste	75.1	71.7	63.1	0.043	73.4	74.7	0.503
3. Muscle pain	65	73.1	56.9	0.002	62.5	69.2	0.001
4. Weakness in the limbs	32.6	41.9	44.6	< 0.001	36.1	33.5	0.192
5. Change in peripheral sensation	21.8	23.7	32.3	0.106	23.3	21.7	0.353
6. Loss of consciousness	17.5	15	32.3	0.003	19.3	16	0.039
7. Seizure	14.2	10.2	24.6	0.004	15.6	12.3	0.024
8. Confusion	21.2	15.5	26.2	0.017	20.3	20.3	0.995
9. A vision change	22.6	24	36.9	0.024	21.6	24.5	0.094
10. An imbalance or gait disturbance	29.8	29.3	33.8	0.757	26.7	32.4	0.003
11. Cerebrovascular accident (stroke)	11.9	9.2	16.9	0.115	13.1	10.3	0.038
12. Difficulty in swallowing	35	31.5	35.4	0.382	34.5	34.3	0.930
13. Mood disturbance	50.2	48.7	43.1	0.474	43.2	54.9	< 0.001

## Discussion

Neurological manifestations associated with COVID-19 are becoming more common. People of different ethnic origins have varying outcomes of COVID-19. Several cohort studies have identified recurrent neurological symptoms and complications related to COVID-19 disease worldwide; the prevalence of neurological manifestations associated with the infection of COVID-19 in Saudi Arabia and other Arab countries is still not determined. Our study attempts to fill this knowledge gap. The results show that the most common neurological manifestations in COVID-19 patients are headache (75.8%), changes in sense of smell and taste (74.1%), muscle pain (66.2%), and mood disturbance (depression, anxiety) (49.7%). These findings are similar to another study when conducting a systematic review of 350 studies in 2021 on recurring neurological manifestations in COVID-19, which showed that the most common neurological symptoms included fatigue (32%), myalgia (20%), impaired taste (21%), impaired sense of smell (19%), and headache (13%) [12].

Headache was found to be the most common and this may be due to the high prevalence of migraine and tension-type headache disorders in Saudi Arabia [13].

Mood disturbance was among the common neurological manifestations which were not assessed with prior similar studies.

Different studies showed conflicting results to ours, which could be due to different ethnic backgrounds and involving participants with comorbidities impacting the prevalence of these symptoms [14,15].

Increased pain sensitivity in women could be responsible for increased muscle pain prevalence among our female participants [16]. Vaccinated participants were found also to have an increased prevalence of different neurological manifestations than those who contracted the virus. Vaccines induce the immune system to produce different inflammatory mediators that could be responsible for causing these symptoms [17].

We figured out that hospitalization and the prevalence of COVID-19-associated neurological symptoms are correlated except for changes in sense of taste and smell and muscle aches.

Atherosclerosis and its complications are more common in men which might be triggering for the increased stroke and its sequelae in this population.

Limitations

A limitation of our study is its retrospective design. Another limitation was that it was small in size. More research is needed to see if similar results are found in larger groups of people.

Other limiting factors that could be affecting the results include voluntarily reported questionnaires; internet-based survey limits responding population; predominantly younger age of responders while neurological complications are seen mostly in the elderly >60 years; the nature of the study excludes those who were dead, or with severe complications resulting in long-term care stay.

## Conclusions

COVID-19 is associated with many neurological manifestations. The prevalence of neurological manifestations associated with this infection in Saudi Arabia is similar to many previous studies done globally. Self-limited symptoms such as headache and change in smell function i.e., anosmia or hyposmia were more pronounced in those <40 years. Acute neurological manifestations such as loss of consciousness and convulsions are seen more in older individuals which may lead to increased mortality and worse outcomes. These directly or indirectly related manifestations are non-specific but mandate more attention, especially to elderly patients with COVID-19, to early detect common neurological manifestations associated with it, and to apply preventive and therapeutic measures known to improve the outcome of these symptoms.
